# Draft Genome Sequence of *Flavobacterium johnsoniae* CI04, an Isolate from the Soybean Rhizosphere

**DOI:** 10.1128/genomeA.01535-16

**Published:** 2017-01-26

**Authors:** Juan I. Bravo, Gabriel L. Lozano, Jo Handelsman

**Affiliations:** Department of Molecular, Cellular and Developmental Biology, Yale University, New Haven, Connecticut, USA

## Abstract

*Flavobacterium johnsoniae* CI04 was coisolated with *Bacillus cereus* from a root of a field-grown soybean plant in Arlington, WI, and selected as a model for studying commensalism between members of the *Cytophaga-Flavobacterium-Bacteroides* group and *B. cereus*. Here we report the draft genome sequence of *F. johnsoniae* CI04 obtained by Illumina sequencing.

## GENOME ANNOUNCEMENT

*Flavobacterium johnsoniae* is a Gram-negative bacterium belonging to the *Cytophaga-Flavobacterium-Bacteroides* (CFB) group and is commonly found in soil and rhizospheres (1, 2). *F. johnsoniae* digests a wide array of complex polysaccharides, but is most recognized for its efficient degradation of insoluble chitin (1), the second most abundant biopolymer on Earth (3). *F. johnsoniae* has been used as a genetic and biochemical model to study CFB gliding motility, a form of translocation that does not appear to share underlying mechanisms of flagellar motility, type IV pilus-mediated twitching motility, or either myxobacterial or mycoplasma gliding motility (4). Moreover, *F. johnsoniae* may be useful for studying the ecological role of this type of motility, which is speculated to give CFBs a competitive advantage by enabling them to translocate to find new carbon and nutrient sources (5). Finally, *F. johnsoniae* is a prospective plant growth-promoting agent, as strain GSE09 produces a volatile compound 2,4-di-tert-butylphenol that inhibits development of the oomycete (protist) pathogen, *Phytophthora capsici*, and the fungal pathogen, *Colletotrichum acutatum*, and stimulates pepper fruit ripening (6, 7). Other *F. johnsoniae* strains produce monobactam and quinolone antibiotics (8–10).

*F. johnsoniae* CI04 was one among 66 bacteria coisolated with *B. cereus* from the roots of field-grown soybean plants (2). These coisolates were bacteria that, after at least 3 days of incubation, grew out of patches of a *B. cereus* culture that had been previously purified by repeated streaking for single colonies. Most of the coisolates were CFB bacteria whose growth was stimulated by *B. cereus in vitro* under conditions that mimic the rhizosphere. *F. johnsoniae* CI04 growth is stimulated by *B. cereus* peptidoglycan, which it appears to degrade with an as-yet-uncharacterized extracellular cell wall-hydrolyzing agent (2).

The *F. johnsoniae* CI04 genome was sequenced using a paired-end approach on the Illumina MiSeq platform. Low-quality sequences were trimmed using Trimmomatic (11) to obtain a total of 7,282,522 pair-reads, which were then assembled into 63 contigs, with a minimum length of 200 bp, using Velvet (12) and VelvetOptimiser (http://bioinformatics.net.au/software.velvetoptimiser). These contigs were ordered by Mauve (13) using the *F. johnsoniae* UW101 genome (14) as a reference. Contigs were assembled manually by joining neighboring sequences with a linker sequence of unknown nucleotide character “N.” Gaps were then filled with GapFiller (15) using the trimmed reads to reduce the number of contigs to 13. The final assembly consisted of 5,492,177 bp in 13 contigs, with an *N*_50_ contig size of 422,475 bp.

We predict that the genome sequence will be useful in probing biocontrol and antibiotic activities of *F. johnsoniae* CI04 and in unraveling the genetic mechanisms driving its interactions with other rhizosphere microbes and plant hosts. These findings may then shed light on the larger ecological role played in the soil by members of the Bacteroidetes phylum, which is poorly understood.

### Accession number(s).

This whole-genome shotgun project has been deposited at DDBJ/ENA/GenBank under the accession no. MLFK00000000. The version described in this paper is the first version.

## References

[1] Stanier RY 1947 Studies on nonfruiting myxobacteria: I. *Cytophaga johnsonae*, n.sp., a chitin-decomposing myxobacterium. J Bacteriol 53:297–315. PMC518309 16561273

[2] Peterson SB, Dunn AK, Klimowicz AK, Handelsman J 2006 Peptidoglycan from *Bacillus cereus* mediates commensalism with rhizosphere bacteria from the Cytophaga-Flavobacterium group. Appl Environ Microbiol 72:5421–5427. doi:10.1128/AEM.02928-05. 16885294 PMC1538759

[3] Muzzarelli RA 1999 Native, industrial and fossil chitins. EXS 87:1–6. 10906948 10.1007/978-3-0348-8757-1_1

[4] McBride MJ 2004 Cytophaga-Flavobacterium gliding motility. J Mol Microbiol Biotechnol 7:63–71. doi:10.1159/000077870. 15170404

[5] Johansen JE, Binnerup SJ 2002 Contribution of Cytophaga-like bacteria to the potential of turnover of carbon, nitrogen, and phosphorus by bacteria in the rhizosphere of barley (*Hordeum vulgare* L.). Microb Ecol 43:298–306. doi:10.1007/s00248-002-2006-z. 12037608

[6] Sang MK, Kim JD, Kim BS, Kim KD 2011 Root treatment with rhizobacteria antagonistic to phytophthora blight affects anthracnose occurrence, ripening, and yield of pepper fruit in the plastic house and field. Phytopathology 101:666–678. doi:10.1094/PHYTO-08-10-0224. 21405997

[7] Sang MK, Kim KD 2012 The volatile-producing *Flavobacterium johnsoniae* strain GSE09 shows biocontrol activity against phytophthora capsici in pepper. J Appl Microbiol 113:383–398. doi:10.1111/j.1365-2672.2012.05330.x. 22563881

[8] Kato T, Hinoo H, Shoji J, Matsumoto K, Tanimoto T, Hattori T, Hirooka K, Kondo E 1987 PB-5266 A, B and C, new monobactams. I. Taxonomy, fermentation and isolation. J Antibiot (Tokyo) 40:135–138. doi:10.7164/antibiotics.40.135. 3570960

[9] Kato T, Hinoo H, Terui Y, Nishikawa J, Nakagawa Y, Ikenishi Y, Shoji J 1987 PB-5266 A, B and C, new monobactams. II. Physico-chemical properties and chemical structures. J Antibiot (Tokyo) 40:139–144. doi:10.7164/antibiotics.40.139. 3570961

[10] Evans JR, Napier EJ, Fletton RA 1978 G1499-2, a new quinoline compound isolated from the fermentation broth of *Cytophaga johnsonii*. J Antibiot (Tokyo) 31:952–958. doi:10.7164/antibiotics.31.952. 711619

[11] Bolger AM, Lohse M, Usadel B 2014 Trimmomatic: a flexible trimmer for illumina sequence data. Bioinformatics 30:2114–2120. doi:10.1093/bioinformatics/btu170. 24695404 PMC4103590

[12] Zerbino DR, Birney E 2008 Velvet: algorithms for *de novo* short read assembly using de Bruijn graphs. Genome Res 18:821–829. doi:10.1101/gr.074492.107. 18349386 PMC2336801

[13] Darling ACE, Mau B, Blattner FR, Perna NT 2004 Mauve: multiple alignment of conserved genomic sequence with rearrangements. Genome Res 14:1394–1403. doi:10.1101/gr.2289704. 15231754 PMC442156

[14] McBride MJ, Xie G, Martens EC, Lapidus A, Henrissat B, Rhodes RG, Goltsman E, Wang W, Xu J, Hunnicutt DW, Staroscik AM, Hoover TR, Cheng YQ, Stein JL 2009 Novel features of the polysaccharide-digesting gliding bacterium *Flavobacterium johnsoniae* as revealed by genome sequence analysis. Appl Environ Microbiol 75:6864–6875. doi:10.1128/AEM.01495-09. 19717629 PMC2772454

[15] Boetzer M, Pirovano W 2012 Toward almost closed genomes with GapFiller. Genome Biol 13:R56. doi:10.1186/gb-2012-13-6-r56. 22731987 PMC3446322

